# Electrochemical Properties and Jet Electrochemical Micromilling of (TiB+TiC)/Ti6Al4V Composites in NaCl+NaNO_3_ Mixed Electrolyte

**DOI:** 10.3390/ma17194904

**Published:** 2024-10-07

**Authors:** Shen Niu, Hao Wang, Pingmei Ming, Ge Qin, Lei Ren, Huan Liu, Xinchao Li

**Affiliations:** School of Mechanical and Power Engineering, Henan Polytechnic University, Jiaozuo 454003, China; haohpu2026@163.com (H.W.); mingpingmei@163.com (P.M.); qinge@hpu.edu.cn (G.Q.); renlei@hpu.edu.cn (L.R.); liuhuan@hpu.edu.cn (H.L.); hpulixinchao@163.com (X.L.)

**Keywords:** (TiB+TiC)/Ti6Al4V composites, jet electrochemical micromilling, electrochemical anodic dissolution, mixed electrolyte, stray corrosion

## Abstract

Difficult-to-cut titanium matrix composites (TiB+TiC)/Ti6Al4V have extensive application prospects in the fields of biomedical and aerospace metal microcomponents due to their excellent mechanical properties. Jet electrochemical micromilling (JEMM) technology is an ideal method for machining microstructures that leverages the principle of electrochemical anodic dissolution. However, the matrix Ti6Al4V is susceptible to passivation during electrochemical milling, and the inclusion of high-strength TiB whiskers and TiC particles as reinforcing phases further increases the machining difficulty of (TiB+TiC)/Ti6Al4V. In this study, a novel approach using NaCl+NaNO_3_ mixed electrolyte for the JEMM of (TiB+TiC)/Ti6Al4V was adopted. Electrochemical behaviors were measured in NaCl and NaCl+NaNO_3_ electrolytes. In the mixed electrolyte, a higher transpassive potential was required to break down the passive film, which led to better corrosion resistance of (TiB+TiC)/Ti6Al4V, and the exposed reinforcing phases on the dissolved surface were significantly reduced. The results of the JEMM machining indicate that, compared to NaCl electrolyte, using mixed electrolyte effectively mitigates stray corrosion at the edges of micro-grooves and markedly improves the uniformity of both groove depth and width dimensions. Additionally, the surface quality was noticeably improved, with a reduction in Ra from 2.84 μm to 1.03 μm and in Rq from 3.41 μm to 1.40 μm.

## 1. Introduction

Titanium matrix composites (TMCs) are a type of metal matrix composite composed of a titanium alloy matrix reinforced with particles, whiskers, fibers, and other strengthening phases [1,2]. Its metal microstructure devices have extensive application prospects in automotive, military, biomedical, aerospace, and other fields owing to their high specific strength, excellent corrosion resistance, and outstanding ductility [3,4,5,6]. For instance, a 2009 NASA study on composites indicated that titanium-based composites accounted for nearly 40% of the F-22 fighter jet, enhancing the corrosion resistance and fatigue strength of the components [7,8]. Additionally, Rolls-Royce designed a composite carbon/titanium fan system for the Advance and UltraFan engines, aiming to reduce fuel consumption and carbon dioxide emissions [9]. Among the various reinforcing phases in TMCs, TiC particles and TiB whiskers have attracted significant attention and research efforts due to their similar density and excellent chemical compatibility with titanium alloy matrices [10]. (TiB+TiC)/Ti6Al4V is a widely utilized type of TMC; the presence of TiC and TiB reinforcing phases can enhance the ductility and Young modulus of the Ti6Al4V matrix [11]. However, (TiB+TiC)/Ti6Al4V is regarded as a typical difficult-to-cut material. Using traditional micro-cutting methods for machining leads to rapid tool wear, stress concentration on the machined surface, low machining accuracy, and poor surface quality [12]. Therefore, micro-machining technology for (TiB+TiC)/Ti6Al4V is increasingly becoming a prominent topic in current precision machining research.

Jet electrochemical micromilling (JEMM) is a micro-electrochemical machining technology that directly uses a metal nozzle as the tool cathode, leveraging the principle of electrochemical anodic dissolution to remove the metal material. Compared to mechanical micromachining, micro-laser processing, and micro-electrical discharge machining, this technology removes material via ion interactions, offering inherent advantages in machining accuracy, material removal rates, and surface quality. Furthermore, it has substantial potential for applications in the field of micro-machining due to its features, including the capability to handle material of varying hardness, minimal tool electrode wear, stress-free machined surfaces, and exceptional flexibility [13]. For instance, Hackert-Oschätzchen et al. [14] used a 0.1 mm inner diameter metal nozzle to fabricate an annular microstructure on a stainless steel surface, achieving dimensions of 180 μm depth and 190 μm width and a surface roughness of 0.1 μm. Luo et al. [15] utilized an array of insulated metal nozzles with a 0.5 mm inner diameter spaced 4 mm apart to obtain densely and intersecting parallel micro-grooves with an average width of 910 μm on the surface of a stainless steel workpiece. Li et al. [16] machined a helical microstructure with an average depth of 102.35 μm, width of 432 μm, and surface roughness of 0.118 μm on a zirconium-based metallic glass specimen using a metal nozzle with an inner diameter of 0.22 mm. Clare et al. [17] employed a metal nozzle with a 0.15 mm inner diameter to create a typical biomedical stent microstructure on the surface of the Inconel 718 workpiece. Lu et al. [18] utilized a metal nozzle with an inner diameter of 0.3 mm to create micro-hole arrays in Ti6Al4V alloy cylinders, demonstrating the potential of JEMM technology in the biomedical field. Guo et al. [19] used a 0.85 mm inner diameter metal nozzle to fabricate micro-grooves on the surface of Alloy 718 that were laser-directed energy deposited, achieving a surface roughness of 0.53 μm. These research papers convincingly demonstrate the feasibility of JEMM technology in the realm of metal micro-machining.

However, due to the propensity of the Ti6Al4V matrix for passivation, the dense passive film that forms on its surface during electrochemical machining impedes the electrochemical dissolution of the material [20]. Additionally, the fragmentation and detachment of TiB and TiC reinforcing phases compromise the surface integrity of the material. This presents further difficulties and challenges in achieving the efficient and high-quality electrochemical machining of (TiB+TiC)/Ti6Al4V. Indeed, anodic electrochemical dissolution properties are critical in electrochemical machining, directly influencing the machining accuracy, surface quality, and processing efficiency of the material. For the electrochemical machining of titanium and titanium alloys, it is standard practice to initially investigate the electrochemical properties of the material in the electrolyte before proceeding with machining. For example, Wang et al. [21] discovered that the TiAl45XD titanium alloy forms a more brittle passive film on its surface in NaCl electrolyte compared to NaNO_3_ electrolyte. This leads to an increased material removal rate during electrochemical machining. They further found that the passive film primarily consists of TiO_2_ and Al_2_O_3_. Compared to NaNO_3_ and NaCl electrolytes, Baehre et al. [22] found that KBr electrolyte can lower the dissolution potential (E_diss_) of intermetallic Ti60Al40; it also increases the maximum current density (J_max_) during the dissolution process and achieves a higher material removal rate. He et al. [23,24] compared the polarization curves and electrochemical dissolution surface morphology of Ti6Al4V in both NaNO_3_ and NaCl electrolytes. They observed that in NaCl electrolyte, the material was more prone to pitting at low current densities and, as the current density increased, the surface quality of the electrochemical machining process was enhanced. Niu et al. [25] noted that using NaCl ethylene glycol-based electrolyte as opposed to NaCl aqueous electrolyte can reduce the stray corrosion of Ti6Al4V. They utilized JEMM technology to fabricate a Ti6Al4V microstructure with high geometric dimensional uniformity.

At present, there is limited research on (TiB+TiC)/Ti6Al4V in the realm of JEMM. Nonetheless, it is worth noting that Li et al. [26,27,28,29] conducted experimental studies on macro electrochemical milling slots of (TiB+TiC)/Ti6Al4V, investigating its electrochemical properties in NaCl and NaNO_3_ electrolytes. They found that using NaNO_3_ electrolyte for electrochemical milling resulted in massive concaves in the slots’ surface, whereas the surface was smoother and flatter with NaCl electrolyte. However, NaCl as a linear electrolyte tends to cause stray corrosion in both non-machined areas and machined surfaces. Therefore, using NaCl electrolyte for the JEMM of (TiB+TiC)/Ti6Al4V impedes achieving high machining accuracy and superior surface quality of microstructures. As noted above, investigation into the electrochemical properties of (TiB+TiC)/Ti6Al4V and JEMM technology is urgently needed.

In this study, the electrochemical dissolution properties of (TiB+TiC)/Ti6Al4V in NaCl+NaNO_3_ mixed electrolyte were innovatively investigated, and JEMM utilizing this mixed electrolyte was applied to (TiB+TiC)/Ti6Al4V for the first time. The electrochemical impedance spectroscopy (EIS), Tafel polarization curves, potentiodynamic polarization curves, and current efficiency of (TiB+TiC)/Ti6Al4V were analyzed in both NaCl and NaCl+NaNO_3_ electrolytes. Furthermore, the dissolution surface morphology was observed under various current densities. Finally, JEMM technology was employed to fabricate rectangular microstructures on the (TiB+TiC)/Ti6Al4V composites. The machining results revealed that the microstructures of (TiB+TiC)/Ti6Al4V produced with the mixed electrolyte achieved greater dimensional accuracy and enhanced surface quality.

## 2. Materials and Methods

### 2.1. Experimental Material

The chemical compositions of the (TiB+TiC)/Ti6Al4V composites used in the experiment are presented in Table 1. The material was prepared into cubic (10 mm × 10 mm × 10 mm), rectangular (5 mm × 5 mm × 10 mm), and thin plate (20 mm × 20 mm × 2 mm) specimens using wire electrical discharge machining (WEDM). One surface of each specimen was polished to a mirror finish using SiC water sandpaper (STARCKE Abrasive Product, Melle, Germany), with grits ranging from 500 to 2000, and ultrasonically cleaned with alcohol for 30 min. These specimens were then used for subsequent electrochemical and current efficiency tests and machining experiments.

### 2.2. Electrochemical Properties Testing

**Figure 1:**
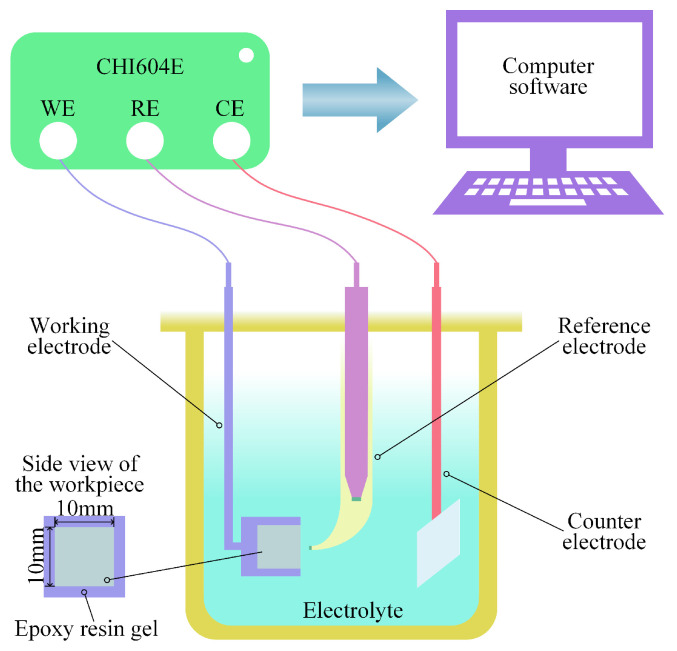
Electrochemical testing experimental equipment diagram.

Electrochemical tests are essential for analyzing the corrosion behavior and dissolution properties of a material, providing both theoretical insights and practical guidance. The EIS, Tafel polarization curves, and potentiodynamic polarization curves of (TiB+TiC)/Ti6Al4V in NaCl and mixed electrolytes were measured using a three-electrode electrochemical workstation (CHI604E, CH Instruments, Shanghai, China); the experimental equipment is shown in Figure 1. In addition, the analytical reagents NaCl and NaNO_3_ were provided by Shanghai Sinopharm Chemical Reagent Co., Ltd. (Shanghai, China) and Tianjin Oubokai Chemical Co., Ltd. (Tianjin, China) respectively. All electrolytes were prepared using distilled water. The electrolyte temperature was maintained at 30 ± 1 °C. The saturated calomel electrode (SCE), workpiece (10mm × 10mm × 10mm), and platinum plate (14.8 mm × 14.8 mm × 0.2 mm) served as the reference electrode (RE), working electrode (WE), and counter electrode (CE), respectively. Only one face of the workpiece was exposed to the electrolyte, with the remaining surfaces covered in epoxy resin. The EIS test was performed with a frequency range of 0.01 Hz to 100,000 Hz and an amplitude of 5 mV; the data were analyzed and fitted using the commercial software ZView2. The Tafel polarization test was conducted with a scan rate of 1 mV/s and an applied potential range of −1 V to −0.35 V. For the potentiodynamic polarization test, a scan rate of 10 mV/s was used, with an applied potential range of −2 V to 10 V. To minimize the randomness of experimental data, each experiment was conducted three times.

### 2.3. Current Efficiency Experiment

**Figure 2:**
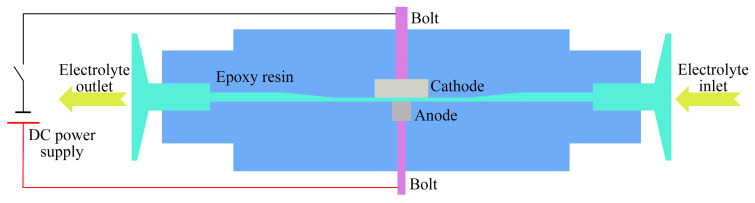
Diagram of the current efficiency measuring experimental equipment.

Current efficiency is a key factor in assessing the performance of metal electrochemical machining. It is defined as the ratio of the actual dissolved mass of a material during electrochemical dissolution to the theoretically calculated dissolved mass. The relationship between the current efficiency and current density of (TiB+TiC)/Ti6Al4V in NaCl and mixed electrolytes at 30 ± 1 °C was measured using the constant current method. The experimental setup is depicted in Figure 2, where the anode is the (TiB+TiC)/Ti6Al4V specimen, the cathode is made of 304 stainless steel, and a bolt provides electrical conduction. To ensure the reliability of the experimental data, each experiment was performed three times. The current density ranged from 0.2 A·cm^−2^ to 64 A·cm^−2^, with a machining gap of 1 mm. Before and after the experiment, each set of specimens was ultrasonically cleaned with alcohol and dried, and weight changes were recorded using a precision analytical balance (SQP, SARTORIUS, Göttingen, Germany). Current efficiency, η, was defined by the following formula:

(1) $$\eta =\frac{M_{1}}{M_{2}}=\frac{M_{1}}{\omega \rho \mathrm{It}}$$

where M_1_ is the actual dissolved mass of the material (g), M_2_ is the theoretical dissolved mass of the material (g), ω is the volume electrochemical equivalent (cm^3^/A·s), ρ is the density of the anode material (g/cm^3^), I is the processing current (A), and t is the processing time (s).

(TiB+TiC)/Ti6Al4V is composed of various metal elements, and its volume electrochemical equivalent, ω, was calculated using the following formula:

(2) $$\omega =\frac{1}{\rho F\left(\frac{n_{1}}{A_{1}}a_{1}+\frac{n_{2}}{A_{2}}a_{2}+\cdots +\frac{n_{j}}{A_{j}}a_{j}\right)}$$

where F is the Faraday constant; n_1_, n_2_, … n_j_ are the valences of the various elements; A_1_, A_2_, … A_j_ are the atomic masses of the elements; and a_1_, a_2_, … a_j_ are the weight percentages of the elements.

Additionally, following the current efficiency tests, the dissolution surface morphology of the samples at different current densities in NaCl and mixed electrolytes was observed and analyzed with a field emission scanning electron microscope (SEM, Merlin Compact, Oberkochen, Germany).

### 2.4. JEMM Experiment

Based on the preliminary exploration of the electrochemical properties of (TiB+TiC)/Ti6Al4V and the initial investigation of JEMM experiments, the decision was made to utilize the experimental parameters outlined in Table 2. Using a multi-pass downward-feeding approach, a rectangular microstructure was machined on the (TiB+TiC)/Ti6Al4V workpiece via JEMM with NaCl and mixed electrolytes. The experimental system used in this study was consistent with that described in [25]. The three-dimensional morphology and surface roughness of the microstructure were measured using a 3D measuring laser microscope (OLS5100, Olympus, Japan), while localized morphology images of the machining profiles were obtained through SEM [30].

## 3. Results and Discussion

### 3.1. EIS Analysis

**Figure 3:**
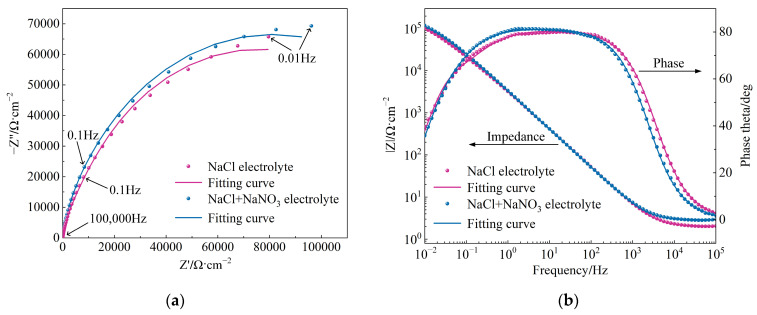
EIS results for (TiB+TiC)/Ti6Al4V in different electrolytes: (**a**) Nyquist plot; (**b**) Bode plot.

Electrochemical impedance refers to the resistance to current flow in an electrochemical system, reflecting a composite response of electrical characteristics such as resistance and capacitance. Figure 3 presents the electrochemical impedance spectroscopy (EIS) test results for (TiB+TiC)/Ti6Al4V in NaCl and mixed electrolytes, shown in both Nyquist and Bode spectra, with the measured data represented as scatter points. In the Nyquist spectra of Figure 3a, the horizontal axis represents the real component and the vertical axis represents the imaginary component of the impedance, respectively. From the figure, it is evident that the Nyquist spectra of (TiB+TiC)/Ti6Al4V in both NaCl and mixed electrolytes displayed similar capacitive arcs, with a larger diameter observed in the mixed electrolyte. This larger diameter indicated greater polarization resistance of the passive film formed on the material’s surface [31]. In the mixed electrolyte, the oxidative properties of NO_3_^−^ reduced the rate of ion exchange at the electrode/solution interface, which increased the resistance of the passive film on the surface of (TiB+TiC)/Ti6Al4V.

The Bode spectra in Figure 3b illustrate the relationship between the magnitude of the impedance |Z|, the phase angle, and the frequency at the electrode surface. From the frequency–impedance spectra, it can be seen that the impedance modulus (|Z|) rose with decreasing scan frequency in both NaCl and mixed electrolytes, attaining its peak value at 10^−2^ Hz. Generally, a higher |Z| value at a frequency near 0 Hz (f = 10^−2^ Hz) signified the superior corrosion resistance of the passive film [32]. At this frequency, the |Z| values in NaCl and mixed electrolytes were 10.32 × 10^4^ and 11.85 × 10^4^, respectively. As previously noted, the presence of NO_3_^−^ enhanced the polarization resistance of the passive film, which, in turn, impeded the charge transfer rate between the electrodes. Consequently, the passive film demonstrated improved corrosion resistance in the mixed electrolyte. However, compared to NO_3_^−^, Cl^−^ had a more pronounced activating effect on the charge transfer between electrodes and the breakdown of the passive film [33]. Furthermore, the NaCl electrolyte had a higher concentration of Cl^−^ than the mixed electrolyte. As a result, the passive film in the NaCl electrolyte exhibited relatively inferior corrosion resistance, leading to easier breakdown under identical current density conditions. From the frequency–phase angle spectra, it can be observed that in both NaCl and mixed electrolytes, the phase angle increased with frequency in the lower frequency range (10^−2^ Hz to 10^0^ Hz). In the mid-frequency range (10^0^ Hz to 10^2^ Hz), the phase angle reached a maximum value and remained relatively constant. This phenomenon indicated the gradual formation of the passive film, which led to the suppression of the charge transfer process between the electrodes [34]. At this point, (TiB+TiC)/Ti6Al4V began to enter the passive stage. In the high-frequency range (10^2^ Hz to 10^5^ Hz), the phase angle decreased with increasing frequency. This characteristic indicated that the passive film formed on the surface began to break down [32]. At this point, the charge transfer rate between the electrodes increased and the matrix transitioned into the electrochemical dissolution stage. Additionally, it can be observed from the figure that within the measured frequency range, the maximum phase angle was less than 90°, indicating that the capacitance in the charge transfer process was non-ideal. Thus, a constant phase element (CPE) was employed to replace the ideal capacitor [35]. These phenomena are consistent with findings in the literature [28]. 

**Figure 4:**
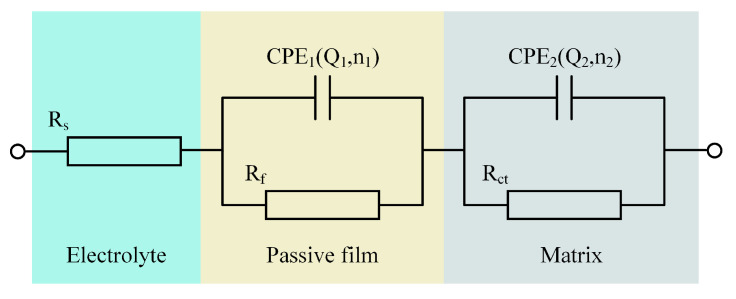
EEC model for the fitting of EIS data.

Using the EIS analysis software Zview2, an equivalent circuit (EEC) model was developed, as shown in Figure 4. The actual measured data were then fitted using this model, with the results depicted by the solid lines in Figure 3. This EEC model comprised electrolyte resistance (R_s_), passive film resistance (R_f_), charge transfer resistance at the electrode/electrolyte interface (R_ct_), and the constant phase elements CPE_1_ and CPE_2_ corresponding to R_f_ and R_ct_, respectively [36]. The impedance of the CPE was calculated using the following equation [37]:

(3) $$Z_{\mathrm{CPE}}=\frac{1}{Q{\left(j\omega \right)}^{n}}$$

where Q is a constant independent of frequency, j is the imaginary unit (j^2^ = −1), ω is the angular frequency (ω = 2πf), and n is the CPE exponent (0 < n < 1), which characterizes the surface inhomogeneity; the closer the n value is to 1, the greater the surface homogeneity [38,39].

Table 3 lists the values of the fitting parameters, where a smaller χ^2^ value signifies a more accurate fit. The value of R_s_ indicated that the mixed electrolyte had greater resistance compared to the NaCl electrolyte. The value of R_f_ showed that the oxidative properties of NO_3_^−^ led to greater resistance of the passive film in the mixed electrolyte, thereby offering stronger protection for the matrix. In the mixed electrolyte, the n_1_ value was closer to 1, suggesting that the passive film was more uniform. The value of R_ct_ revealed the difficulty of charge transfer at the electrode/electrolyte interface. Due to the lower reactivity of NO_3_^−^ relative to Cl^−^, the R_ct_ value was higher in the mixed electrolyte, resulting in increased resistance to charge transfer. As mentioned above, using the mixed electrolyte for the JEMM of (TiB+TiC)/Ti6Al4V provided better protection to the non-machined areas of the microstructure and reduced the impact of stray corrosion on machining quality.

### 3.2. Anodic Polarization Curves

**Figure 5:**
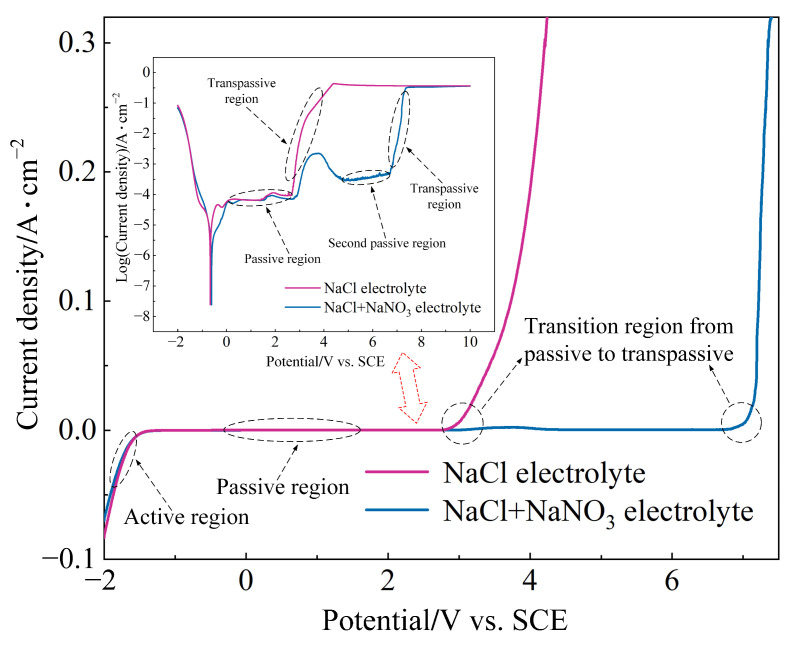
Potentiodynamic polarization curves of (TiB+TiC)/Ti6Al4V in different electrolytes.

As shown in Figure 5, the potentiodynamic polarization curves clearly reveal the active–passive–transpassive polarization characteristics of (TiB+TiC)/Ti6Al4V in both NaCl and mixed electrolytes. It can be observed from the figure that in the active region, the current density slightly increased from negative values, indicating that the electrochemical dissolution reaction of the matrix was gradually activated. As the applied potential increased, the current density remained stable over a wide potential range, implying that the passive film began to form on the surface. During this period, the dissolution process of the matrix was inhibited and (TiB+TiC)/Ti6Al4V entered the passive stage. In NaCl electrolyte, the high activating nature of Cl^−^ decreased the polarization resistance of the passive film, accelerated the ion exchange rate at the electrode/electrolyte interface, and made the passive film more prone to breakdown, resulting in a shorter passive time. When the potential increased to around 2.7 V, the current density suddenly increased sharply, suggesting that the passive film had broken down, and the matrix began to undergo intense electrochemical dissolution, with (TiB+TiC)/Ti6Al4V entering the transpassive stage. However, in comparison to NaCl electrolyte, the mixed electrolyte had a lower concentration of activating Cl^−^, and the oxidative properties of NO_3_^−^ led to a passive film with greater polarization resistance and better corrosion protection. As a result, the passive film was less likely to undergo breakdown, leading to a relatively longer passive time.

It is noteworthy that in the mixed electrolyte, after a brief period of transpassivity, the current density sharply decreased. This indicated that the passive film started to reform on the surface, thereby inhibiting the electrochemical dissolution process of the matrix and leading to a secondary passive phase of (TiB+TiC)/Ti6Al4V. The oxidative capability of NO_3_^−^ in the mixed electrolyte resulted in a higher passive film growth rate than the breakdown rate during the transpassive stage. Therefore, the secondary passive phase occurred only in the mixed electrolyte, unlike in the NaCl electrolyte. When the applied potential reached approximately 6.72 V, the current density sharply increased again. This signaled that the newly formed passive film broke down once more, returning (TiB+TiC)/Ti6Al4V to the transpassive stage, where the electrochemical dissolution of the matrix resumed. In the related literature, (TiB+TiC)/Ti6Al4V also exhibited a secondary passive phase [26].

**Figure 6:**
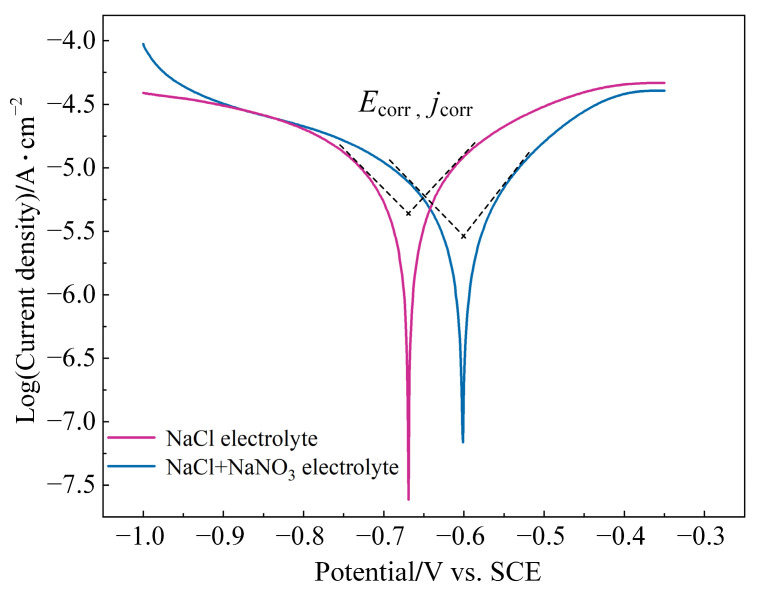
Tafel polarization curves of (TiB+TiC)/Ti6Al4V in different electrolytes.

Figure 6 illustrates the Tafel polarization curves for (TiB+TiC)/Ti6Al4V in both NaCl and mixed electrolytes. From the figure, the corrosion potential (E_corr_) and corrosion current density (j_corr_) can be determined using the Tafel extrapolation method [40], as shown in Table 4. Generally, a higher E_corr_ value indicated better corrosion resistance of the material [41]. According to Faraday’s law, j_corr_ is directly proportional to the electrochemical dissolution rate. As noted from the EIS analysis in Section 3.1, the oxidative properties of NO_3_^−^ increased the polarization resistance of the passive film and slowed down the ion exchange rate at the electrode/electrolyte interface. Thus, in the mixed electrolyte, (TiB+TiC)/Ti6Al4V exhibited a higher E_corr_ value and better corrosion resistance. However, compared to NaCl electrolyte, the lower concentration of activating Cl^−^ in the mixed electrolyte resulted in a slower electrochemical dissolution rate.

As previously discussed, the high concentration of Cl^−^ in NaCl electrolyte made the passive film more susceptible to breakdown, resulting in poorer corrosion resistance for the (TiB+TiC)/Ti6Al4V. This caused the matrix to remain in a state of intense electrochemical dissolution during the transpassive stage, which impaired the accuracy of machining in JEMM. Therefore, using NaCl electrolyte in the JEMM of (TiB+TiC)/Ti6Al4V may impact the dimensional accuracy of the microstructure. In the mixed electrolyte, when the applied potential was approximately 6.72 V, (TiB+TiC)/Ti6Al4V entered the transpassive state, indicating improved corrosion resistance. Additionally, the secondary passive phenomenon helped mitigate intense matrix dissolution during the transpassive stage, as the oxidative effect of NO_3_^−^ and the activating effect of Cl^−^ offset each other, preventing excessive corrosion. Therefore, when using mixed electrolytes for the JEMM of (TiB+TiC)/Ti6Al4V, it is possible to ensure a controlled dissolution rate of the matrix while also improving the dimensional accuracy of the microstructure.

### 3.3. Current Efficiency

**Figure 7:**
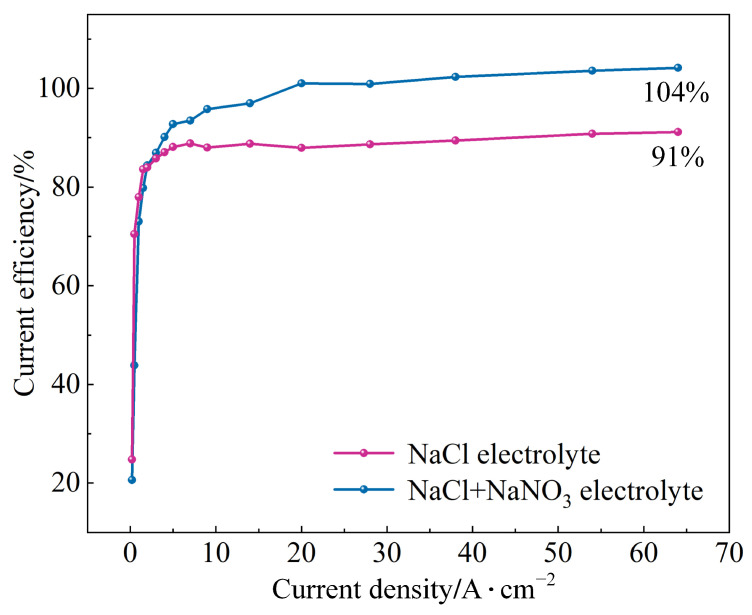
Current efficiency curves of (TiB+TiC)/Ti6Al4V in different electrolytes.

The current efficiency curves of (TiB+TiC)/Ti6Al4V in NaCl and mixed electrolytes are shown in Figure 7, with a current density range of 0.2–64 A·cm^−2^. It can be observed that in both electrolytes, the current efficiency initially increased sharply and then gradually stabilized. At a low current density, the current efficiency of both was very similar. However, when the current density exceeded 5 A·cm^−2^, the current efficiency of (TiB+TiC)/Ti6Al4V in the mixed electrolyte was significantly higher. According to the literature, during the electrochemical machining of (TiB+TiC)/Ti6Al4V, using NaNO_3_ electrolyte results in a greater number and larger size of by-product clusters adhering to the matrix surface compared to NaCl electrolyte. These larger clusters can enclose more undissolved matrix particles. When these by-products are flushed away by the flowing electrolyte, the enclosed undissolved particles are also removed, leading to an increase in the actual calculated current efficiency [26]. Therefore, in this experiment, using NaCl+NaNO_3_ mixed electrolyte resulted in a higher actual calculated current efficiency for (TiB+TiC)/Ti6Al4V in comparison with NaCl electrolyte. Ultimately, when the current density reached 64 A·cm^−2^, the current efficiency was 91% for NaCl electrolyte and 104% for the mixed electrolyte. Similarly, for the substrate Ti6Al4V, the current efficiency in 10% NaNO_3_ electrolyte also exceeded 100%, reaching 134% when the current density increased to 43.75 A·cm^−2^ [42]. Additionally, the current efficiency of (TiB+TiC)/Ti6Al4V in NaNO_3_ electrolyte was higher than that in NaCl electrolyte, recording values of 96.8% and 87.5% at a current density of 50 A·cm^−2^ [26].

**Figure 8:**
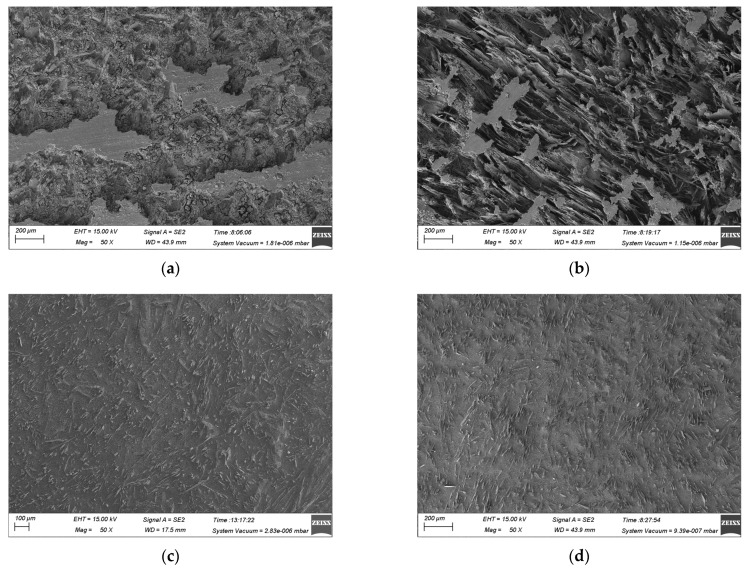
Dissolution surface morphology of (TiB+TiC)/Ti6Al4V in NaCl electrolyte at different current densities: (**a**) 1.52 A·cm^−2^; (**b**) 5 A·cm^−2^; (**c**) 28 A·cm^−2^; (**d**) 64 A·cm^−2^.

**Figure 9:**
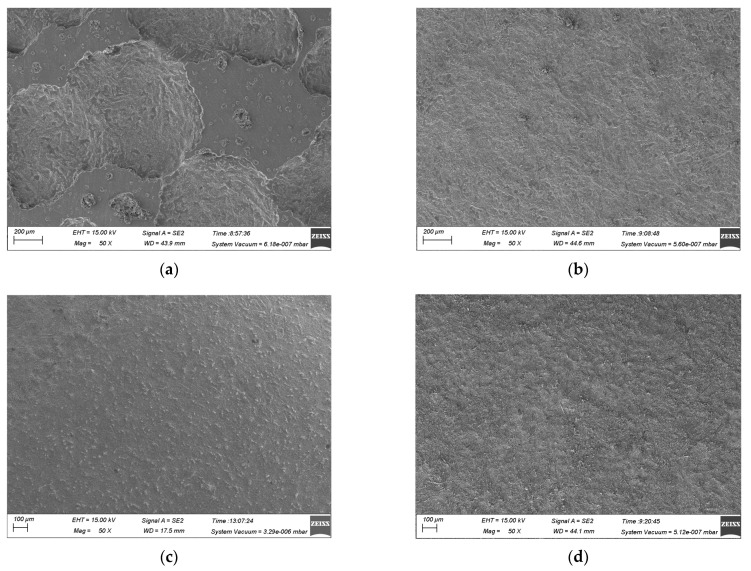
Dissolution surface morphology of (TiB+TiC)/Ti6Al4V in NaCl+NaNO_3_ electrolyte at different current densities: (**a**) 1.52 A·cm^−2^; (**b**) 5 A·cm^−2^; (**c**) 28 A·cm^−2^; (**d**) 64 A·cm^−2^.

The surface dissolution surface morphology of (TiB+TiC)/Ti6Al4V at different current densities in NaCl and mixed electrolytes was observed using SEM, as depicted in Figure 8 and Figure 9. Based on the EIS analysis in Section 3.1 and the polarization curves in Section 3.2, it is evident that the activating effect of Cl^−^ could cause the passive film to break down at a low current density, making the matrix more susceptible to entering the electrochemical dissolution stage. In NaCl electrolyte, the high concentration of Cl^−^ accelerated the breakdown rate of the passive film, thereby intensifying the electrochemical dissolution reaction. Analysis of the dissolution surface morphology at a current density of 1.52 A·cm^−2^ and 5 A·cm^−2^ showed that under a low current density, the matrix underwent uneven dissolution, causing a rough surface with pits and protrusions (Figure 8a). The low current density was insufficient to cause substantial dissolution of the matrix, leaving the embedded reinforcement phases only partially exposed and still integrated with the surrounding undissolved matrix. This led to a poor dissolution surface morphology (Figure 8b). However, in the mixed electrolyte, the enhanced corrosion resistance of (TiB+TiC)/Ti6Al4V and the lower concentration of Cl^−^ further reduced matrix involvement in the electrochemical dissolution reaction. As a result, under the same current density conditions, the reinforcement phases were exposed to a lesser extent, which helped achieve a smoother dissolution surface (Figure 9a,b).

As the current density increased, the electrochemical dissolution effect was enhanced, resulting in more matrix involvement in the dissolution process and a subsequent increase in the by-products produced during electrolysis. According to the literature, compared to NaCl electrolyte, NaNO_3_ electrolyte leads to more undissolved matrix particles being carried away with by-product clusters by the flowing electrolyte. Additionally, the reinforcement phases do not engage in the electrochemical reaction; instead, they detach under the flushing action of the electrolyte as the surrounding matrix is removed [26,27]. Therefore, in the mixed electrolyte, more reinforcement phases detached due to the loss of support from the surrounding matrix. Examination of the SEM images at current densities of 28 A·cm^−2^ and 64 A·cm^−2^ revealed that at a higher current density, the dissolved surface in NaCl electrolyte was densely covered with exposed reinforcement phases (Figure 8c,d). In contrast, in the mixed electrolyte, the exposed reinforcement phases on the dissolved surface were significantly reduced, and the surface morphology was noticeably improved (Figure 9c,d). Overall, relative to NaCl electrolyte, using the mixed electrolyte for the JEMM of (TiB+TiC)/Ti6Al4V was beneficial for enhancing the surface quality of the microstructure.

### 3.4. JEMM of (TiB+TiC)/Ti6Al4V Microstructure

**Figure 10:**
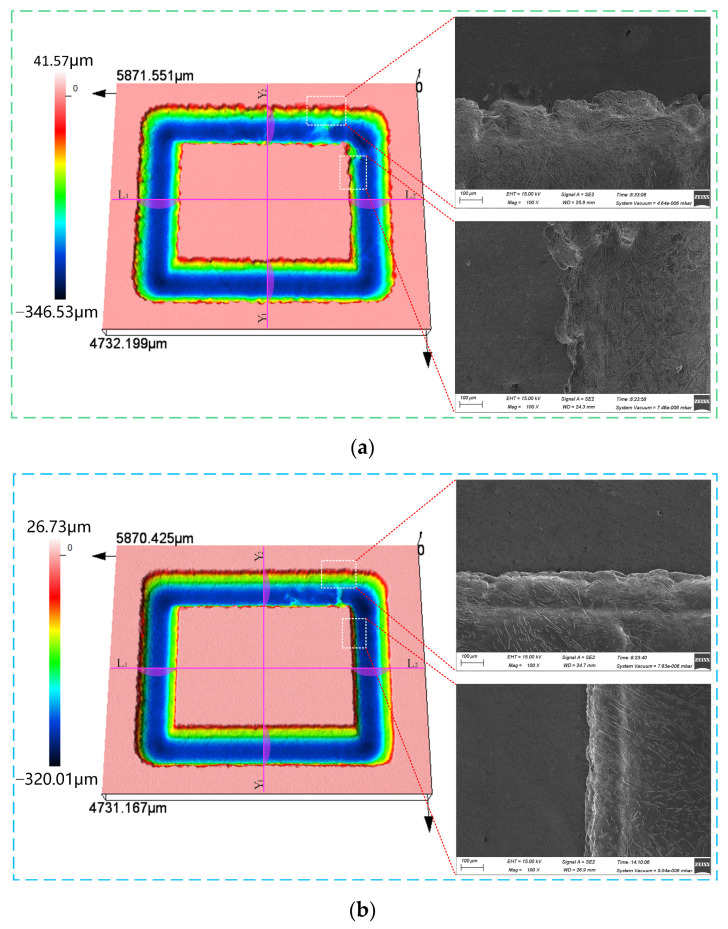
Rectangular microstructure machined by JEMM in different electrolytes: (**a**) NaCl electrolyte; (**b**) NaCl+NaNO_3_ electrolyte.

**Figure 11:**
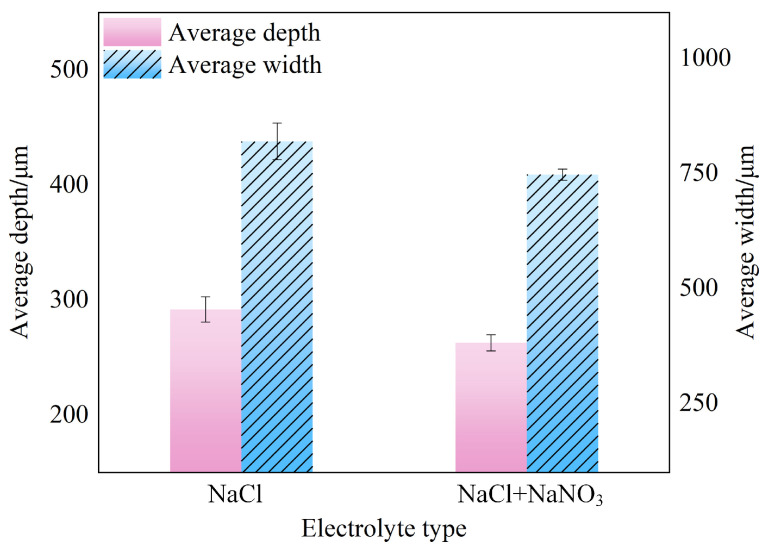
Average depth and width of microstructure machined in different electrolytes.

**Figure 12:**
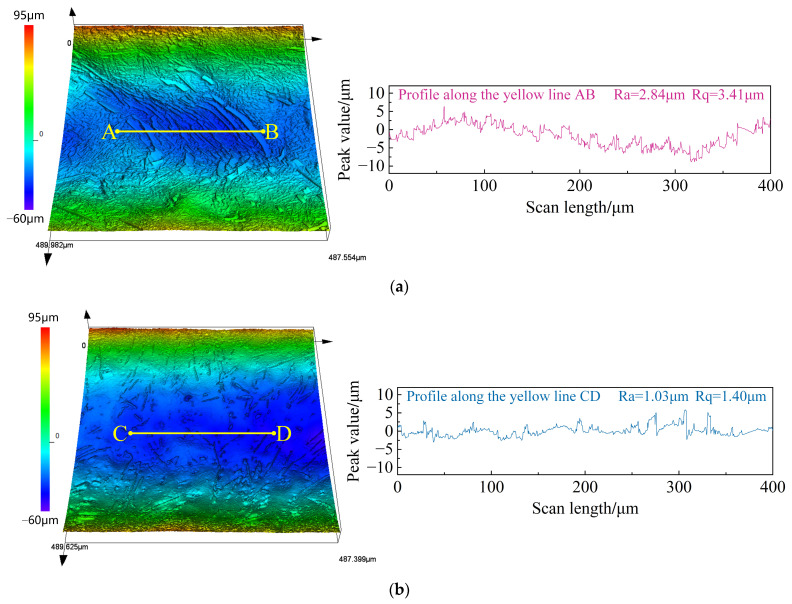
Surface morphology and surface roughness of microstructure machined in different electrolytes: (**a**) NaCl electrolyte; (**b**) NaCl+NaNO_3_ electrolyte.

As illustrated in Figure 10a, the average groove depth and width of the rectangular microstructure machined with NaCl electrolyte in the L_1_–L_2_ and Y_1_-Y_2_ directions were 291.8 μm and 819.6 μm, with standard deviations of 2.66 μm and 3.50 μm, respectively. From the localized SEM images (100×), it can be observed that the micro-groove edges exhibited a serrated appearance, with poor dimensional uniformity and significant stray corrosion. In contrast, as depicted in Figure 10b, the use of the mixed electrolyte significantly improved the dimensional accuracy of the microstructure compared to the NaCl electrolyte. The average groove depth and width in the L_1_–L_2_ and Y_1_-Y_2_ directions were 262.7 μm and 747.4 μm, with standard deviations of 1.48 μm and 0.66 μm, respectively. From the local SEM images (100×), it can be observed that the morphology of the micro-groove edges was markedly enhanced. Figure 11 presents the average groove depth and width of the microstructure machined using NaCl and mixed electrolyte. Additionally, Figure 12 displays the three-dimensional surface morphology of the micro-groove bottom. As shown in Figure 12a, a pronounced distribution of densely packed reinforcement phases was distinctly observed on the material surface when machined with NaCl electrolyte. However, as demonstrated in Figure 12b, fewer reinforcement phases were sparsely distributed, and the surface was smoother with the use of the mixed electrolyte. Simultaneously, the Ra measured for segment AB was 2.84 μm and the Rq was 3.41 μm, compared to the Ra of 1.03 μm and the Rq of 1.40 μm for segment CD. The above analysis reveals that using the mixed electrolyte in JEMM achieved (TiB+TiC)/Ti6Al4V microstructures with higher dimensional accuracy and improved surface quality.

## 4. Conclusions

In this study, the electrochemical dissolution properties of (TiB+TiC)/Ti6Al4V composites reinforced with TiB and TiC were investigated in NaCl and NaCl+NaNO_3_ mixed electrolytes and the dissolution surface morphology at various current densities was observed through current efficiency tests. The rectangular microstructure of (TiB+TiC)/Ti6Al4V was fabricated via JEMM. The conclusions can be summarized as follows.

The electrochemical properties of (TiB+TiC)/Ti6Al4V in NaCl electrolyte and NaCl+NaNO_3_ mixed electrolyte were investigated. Electrochemical impedance spectroscopy and polarization curves revealed that compared to the NaCl electrolyte, the oxidative effect of NO_3_^−^ in the mixed electrolyte led to higher values of electrochemical impedance, passive film resistance, and corrosion potential for (TiB+TiC)/Ti6Al4V. This indicates that a higher transpassive potential was needed to break down the passive film, implying improved corrosion resistance of (TiB+TiC)/Ti6Al4V.

The current efficiency test results indicate that in comparison with NaCl electrolyte, the enhanced corrosion resistance of (TiB+TiC)/Ti6Al4V in the mixed electrolyte reduced matrix involvement in the dissolution reaction at a low current density. As a result, the reinforcement phases were exposed to a lesser extent, leading to a smoother dissolved surface. Under high current density conditions, more reinforcement phases lost support from the surrounding matrix in the mixed electrolyte and detached due to the electrolyte flow. This led to a significant reduction in the exposure of reinforcement phases on the dissolved surface and markedly improved surface morphology.

The JEMM results reveal that compared to NaCl electrolyte, the mixed electrolyte enhanced the transpassive potential of (TiB+TiC)/Ti6Al4V, effectively reducing stray corrosion at the edges of micro-grooves. Therefore, the dimensional uniformity of both groove depth and width was improved. Additionally, due to the reduced exposure of reinforcement phases on the dissolved surface in the mixed electrolyte, the surface quality of the micro-grooves was noticeably enhanced, with reductions in Ra and Rq from 2.84 μm to 1.03 μm and 3.41 μm to 1.40 μm, respectively.

## Figures and Tables

**Table 1:** Chemical composition of (TiB+TiC)/Ti6Al4V composites.

Element	H	N	C	O	Fe	V	Al	Ti	TiB	TiC
Content (wt%)	0.014	0.046	0.092	0.184	0.275	3.671	5.507	81.996	6.470	1.745

**Table 2:** Parameters of the JEMM experiment.

Machining Condition	Parameter
Electrolyte	10% NaCl 5% NaCl + 5% NaNO_3_
Electrolyte temperature (°C)	30 ± 1
Electrolyte pressure (Mpa)	1
Applied voltage (V)	25
Nozzle inner diameter (mm)	0.36
Nozzle outer diameter (mm)	0.63
Initial machining gap (μm)	200
Feed rate (μm/s)	100

**Table 3:** EEC model fitting parameters for (TiB+TiC)/Ti6Al4V in different electrolytes.

Electrolyte	R_s_(Ω·cm^−2^)	Q_1_ × 10^−5^ (Ω^−1^S^n^cm^−2^)	n_1_	R_f_ (Ω·cm^−2^)	Q_2_ × 10^−5^ (Ω^−1^S^n^cm^−2^)	n_2_	R_ct_ (KΩ·cm^−2^)	Chi-Squared ×10^−2^
NaCl	2.033	21.121	0.891	5742	8.192	0.903	142.42	0.26027
NaCl+NaNO_3_	2.883	35.172	0.949	5868	6.762	0.9	153.38	0.57138

**Table 4:** Corrosion parameters of (TiB+TiC)/Ti6Al4V obtained from Tafel polarization curves.

Electrolyte	β_a_ (V/decade)	β_c_ (V/decade)	R_p_ (Ω·cm^−2^)	E_corr_ (mV/SCE)	J_corr_ (μA·cm^−2^)
NaCl electrolyte	0.474	0.52057	5626	−669	8.23
NaCl+NaNO_3_ electrolyte	0.786	0.394	13683	−616	3.48

## Data Availability

Data are contained within the article.
